# Measuring What Matters for Breast Cancer Survivors: Translation, Cross-Cultural Adaptation and Validation of the Croatian Version of Lymphedema Quality of Life Tool-Arm

**DOI:** 10.3390/jcm15020465

**Published:** 2026-01-07

**Authors:** Ivana Klarić-Kukuz, Ana Ćurković, Josipa Grančić, Jure Aljinović, Blaž Barun, Dinko Pivalica, Ana Poljičanin

**Affiliations:** 1 Faculty of Health Sciences, University of Split, 21000 Split, Croatia; ivanaklaric.k@gmail.com (I.K.-K.); josipa.grancic@gmail.com (J.G.); jure.aljinovic@mefst.hr (J.A.);; 2 Institute of Physical Medicine and Rehabilitation with Rheumatology, University Hospital of Split, 21000 Split, Croatia; blaz.barun@mefst.hr; 3 School of Medicine, University of Split, 21000 Split, Croatia

**Keywords:** quality of life, breast neoplasms, lymphedema, rehabilitation, patient-reported outcomes, validation

## Abstract

**Background:** Breast cancer-related lymphedema is a common long-term complication of breast cancer treatment that affects physical functioning, emotional well-being, and quality of life. Although the Lymphedema Quality of Life Questionnaire-Arm (LYMQoL-Arm) is widely used internationally, no Croatian version has been available. The primary objective of this study was to translate and validate the Lymphedema Quality of Life Questionnaire-Upper Limb-Croatian (LYMQoL-UL-CRO) version and evaluate its psychometric properties. A secondary objective was to examine associations between its scores and the relative volume change (RVC) of the affected limb to assess construct validity further. **Methods:** A retrospective cross-sectional study was conducted in 87 women at least six months post-treatment. The questionnaire was translated using a forward-backward procedure. Participants completed the LYMQoL-UL-CRO, the Short Form-36 Health Survey (SF-36), Pain Intensity Numerical Rating Scale, and underwent clinical examination and limb-volume assessment. Test–retest reliability was assessed in 68 participants after 10 days. Psychometric analyses included internal consistency, intraclass correlation coefficients, measurement error indices, construct and discriminant validity tests, exploratory factor analysis, and evaluation of floor and ceiling effects. **Results:** LYMQoL-UL-CRO domains demonstrated acceptable to strong internal consistency and moderate test–retest reliability, with low measurement error. Strong negative correlations with the SF-36 Physical Component Summary supported construct validity, and participants with RVC ≥ 5% reported worse scores, supporting discriminant validity. Exploratory factor analysis confirmed the original four-factor structure, and no floor or ceiling effects were observed. **Conclusions:** The LYMQoL-UL-CRO is a reliable, valid, and culturally appropriate tool for assessing quality of life in Croatian breast cancer survivors with upper-limb lymphedema.

## 1. Introduction

Advancements in breast cancer treatment over the past two decades have significantly increased long-term survivorship [1]. Survivorship phase in cancer care continuum, beginning five years after completion of treatment, marks a transition in patient care as oncologic follow-up becomes less frequent, and survivors face late treatment-related toxicities, new comorbidities, and ongoing psychosocial challenges [2].

However, existing clinical practice guidelines largely address short-term side effects, within five years post-treatment. Evidence on outcomes beyond this period remains limited, creating a substantial gap in knowledge and care for long-term survivors [2,3]. This gap leaves long-term survivors without sufficient guidance for managing persistent symptoms, comorbidities, and psychological issues. Long-term breast cancer survivors experience physical symptoms such as fatigue, chronic pain, lymphedema, and sleep disturbance as well as psychological problems such as anxiety and depression, which may negatively impact quality of life (QoL) and may require specialised care [4]. Although guidelines are meant to be updated every three to five years, many fail to incorporate the most recent evidence and recommendations relevant to the long-term survivorship phase. Consequently, research and clinical frameworks still focus mainly on recurrence surveillance and physical outcomes, with less attention to comprehensive survivorship care that supports overall well-being and quality of life [2,5].

Recent literature increasingly supports the view of breast cancer as a chronic condition, necessitating ongoing surveillance and long-term management comparable to other chronic diseases, such as hypertension or diabetes [6,7,8]. Compared with other chronic condition guidelines, which offer clear, structured instructions on nutrition or physical activity, recommendations for long-term breast cancer survivors remain limited and poorly defined. Existing guidelines are generally broad and lack specificity regarding the particular breast cancer stage or survivorship they meant to address. This perspective challenges the notion that breast cancer is fully resolved after primary treatment, as both late treatment side effects and disease-related sequelae often persist and substantially influence survivors’ long-term quality of life [9,10].

Breast cancer-related lymphedema (BCRL) is among the most common and distressing complications affecting approximately 30% of breast cancer survivors, usually within the first two years following treatment [11]. It results from damage or obstruction caused by surgery or radiation, leading to impaired lymphatic drainage, chronic inflammation, and persistent swelling [12]. Beyond physical symptoms such as swelling, heaviness, tightness, discomfort, pain, dermatological alterations, BCRL often cause functional limitations, body image disorders, and psychological distress, collectively contributing to reduced health-related quality of life (HRQoL) [9,10,13,14,15,16,17,18]. The severity and combination of these symptoms vary widely among individuals [19]. Although most of the studies focus on identifying upper limb symptoms and factors contributing to the development of BCRL, there remains a limited understanding of the complex interrelationships among lymphedema symptoms, knowledge crucial for improving patient care and quality of life [20,21,22,23].

Patient-reported outcome measures (PROMs) are increasingly used in clinical practice to assess HRQoL among lymphedema patients, where they capture patients’ subjective experience and complement objective clinical indicators [22,24,25]. In BCRL, assessing HRQoL is particularly important due to the multidimensional impact across physical, psychological, and social domains. While generic QoL questionnaires used to evaluate BCRL patients’ outcomes [26]. provide valuable insights about general aspects of survivorship, they often fail to capture the specific and multifaceted challenges associated with BCRL, such as functional limitations, body image concerns, and emotional distress. To address these limitations, disease-specific instruments have been developed to more accurately capture symptom burden and functional impact [27,28,29,30].

One of the most widely used tools regarding this manner is the Lymphedema Quality of Life Questionnaire (LYMQoL), designed to assess upper and lower limb lymphedema [26,31,32]. Originally developed in English, it has been culturally adapted and validated in several countries, including Italy, Sweden, Turkey, the United Kingdom, Korea, China, and the Netherlands. Across these diverse populations, the instrument has demonstrated strong psychometric properties, supporting its utility for both clinical care and research [26,29,33,34,35,36,37,38,39]. LYMQoL enables a nuanced and multidimensional evaluation of patient-reported outcomes across Appearance, Function, Symptom, and Mood domains, thereby guiding personalised care and interventions aimed at improving HRQoL among women with BCRL [9,32,40]. However, findings suggest that LYMQoL scores do not always correlate with objective clinical measures such as limb volume, suggesting potential limitations in construct validity [29,41].

Despite the widespread international use of LYMQoL, no validated Croatian version of the instrument exists for women with BCRL. Considering that lymphedema assessment should encompass not only objective measurements but also self-reported experiences, the adaptation of a culturally appropriate, disease-specific tool is essential [11,13,14,18,42,43,44]. The availability of such an instrument would enable more accurate evaluation of patients’ needs, enable monitoring of treatment outcomes, and ultimately support patient-centred care in Croatia.

Therefore, the primary aim of this study was to translate and validate the Croatian version of the LYMQoL for upper limb lymphedema, and to examine its psychometric properties with respect to validity, reliability, and factor structure in a population of Croatian breast cancer survivors with BCRL. The secondary aim was to explore the relationship between LYMQoL-UL-CRO (Lymphedema Quality of Life-Upper Limb-Croatian) scores and objective clinical indicators of lymphedema (upper limb volume difference) to assess the construct validity of the questionnaire and to better understand the association between self-reported quality of life and clinical measures.

## 2. Materials and Methods

### 2.1. Ethical Approval

The study protocol was approved by the Ethical Committee of the University Hospital Split (protocol code 2181-147/01/06/LJ.Z.-23-2) on 28 February 2023. This study was conducted in accordance with the principles of the Declaration of Helsinki. Before enrolment in the study, all participating women were informed about the nature of the study, and written informed consent was obtained.

### 2.2. Study Population

**Figure 1:**
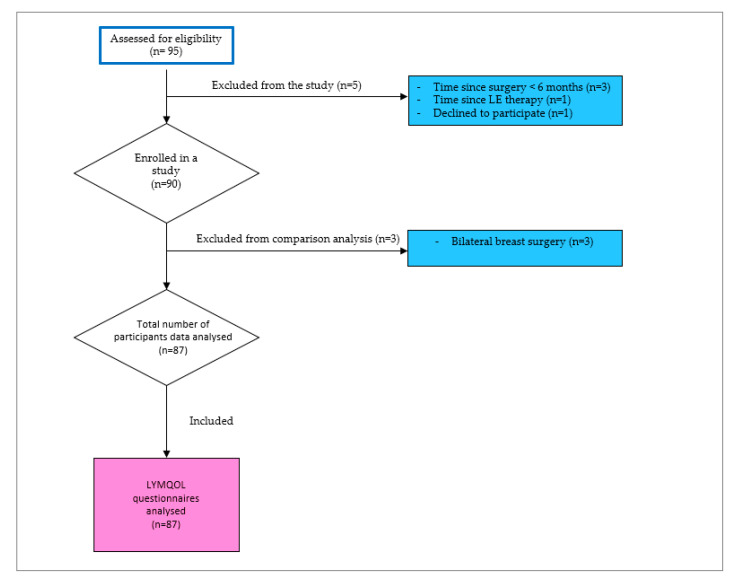
Flowchart of participants’ enrolment. White boxes indicate stages of participant assessment and inclusion, blue boxes indicate reasons for exclusion, and the pink box indicates the final sample included in the LYMQOL questionnaire analysis.

A total of 95 breast cancer survivors participated in this prospective observationalcross-sectional study conducted between 1 November 2023 to 30 March 2024. Participants were recruited from the Department of Physical Medicine, Rehabilitation, with Rheumatology at University Hospital Split, specifically among breast cancer survivors attending follow-up and treatment at the Lymphedema Daily Clinic. The eligibility criteria of the study population were: women aged 18 years or older who had completed treatment for unilateral breast cancer at least six months before study enrolment. The exclusion criteria were: women with bilateral mastectomy or metastatic breast cancer, cognitive impairments, pre-existing arm lymphedema before the initiation of breast cancer treatment, and active lymphedema treatment within three months before enrolment. Following the application of these exclusion criteria, data from 8 participants were excluded from the final analysis. Therefore, the final analysis was conducted on 87 breast cancer survivors (Figure 1).

### 2.3. Lymphedema Diagnosis

#### 2.3.1. Volume Measurements of the Upper Limbs

The volume of the upper limbs was determined using a mathematical formula to calculate the relative difference in volume between the affected and the contralateral upper limb [44,45,46]. The standardised protocol used was described in detail in a previously published study [47].

In the present analysis, we adopted a lower threshold for relative volume change (RVC ≥ 5%) to enable the detection of early clinical lymphedema [13,48,49,50,51,52]. Although an RVC ≥ 10% is commonly used as the diagnostic criterion for clinically manifest BCRL, evidence suggests that smaller but measurable increases in arm volume may already reflect early pathophysiological changes [45,47,48,49,51]. To capture both stages, patients were classified into two groups: those with smaller volume differences (RVC < 5%), indicating subclinical BCRL, and those with larger volume differences (RVC ≥ 5%), indicating early clinical BCRL [46,53]. All of the participants (*n* = 87) completed the Lymphedema Quality of Life Questionnaire-Upper Limb-Croatian (LYMQoL-UL-CRO), Short-Form Health Survey with 36 Items (SF-36), and Pain Intensity Numerical Rating Scale (PINRS) questionnaires on the day of limb volume assessment.

#### 2.3.2. Patients’ Self-Perceived Lymphedema

As in previous research, self-perceived BCRL was classified based on participants’ responses to a standardised question asked by the researcher, “At the moment, does your arm at the side of the surgery feel swollen?” A positive response was classified as the presence of BCRL, whereas a negative response was classified as its absence [54,55]. Women who reported self-perceived BCRL were further investigated regarding the perceived arm swelling (mild, moderate, or severe) [54,55].

### 2.4. Instruments/Outcome Measures

#### 2.4.1. Lymphedema Quality of Life Questionnaire-Arm (LYMQoL-Arm)

The LYMQoL—Arm questionnaire was developed by Keeley et al. in 2010 as a disease-specific instrument for assessing health-related quality of life (HRQoL) in patients with lymphedema [26]. It is a self-administered tool comprising 21 items. The first 20 items assess the impact of lymphedema on HRQOL across four domains: function, appearance, symptoms, and mood. Specifically, the function domain includes items 1–3, with item 1 further divided into eight sub-items (1a to 1h); the Appearance domain comprises items 4–8; the Symptoms domain covers items 9–14; and the Mood domain comprises items 15–20. Each item is rated on a four-grade Likert-like scale as Not at all (1); A little (2); Quite a bit (3) or A lot (4), with higher scores indicating lower HRQoL. Domain scores are calculated by summing the responses within each domain and dividing by the number of items answered. The four domain-specific summary scores are then analysed collectively. If more than 50% of the items in a domain were left unanswered, the corresponding domain score is assigned a value of zero. The final item (item 21) assesses global HRQoL, on an 11-point numerical rating scale ranging from 0 (poor HRQoL) to 10 (excellent HRQoL). For this study, we administered the translated and culturally adopted Croatian version of the LYMQoL questionnaire, Lymphedema Quality of Life Questionnaire-Upper Limb- Croatian (LYMQOL-UL-CRO) [26].

#### 2.4.2. Short-Form Health Survey with 36 Items (SF-36)

Assessing criterion validity requires comparison with a recognised gold standard. In most studies, authors have relied on the SF-36 questionnaire [29,33]. The SF-36 is a self-reported measure of general health-related quality of life (HRQoL), an internationally recognised questionnaire, with a validated Croatian version available [54,56]. It comprises eight distinct scales. The Physical Component Summary (PCS) of HRQoL includes Physical Functioning (10 items), Bodily Pain (2 items), Role Limitations due to Physical Health (4 items), and General Health Perceptions (5 items). These domains collectively assess the physical aspects of HRQoL. The Mental Component Summary (MCS) includes Vitality (4 items), Social Functioning (2 items), Role Limitations due to Emotional Problems (3 items), and Mental Health (5 items), capturing the psychological and social dimensions of HRQoL [56]. For this study, we administered the Croatian version of the SF-36. Scoring was conducted following a standardised 3-step procedure, in accordance with the User’s Manual of the Croatian version [56]. To derive the two higher-order summary scores: Physical Component Summary (PCS) and Mental Component Summary (MCS) [56,57]. The final score ranges between 0 and 100. In contrast to LYMQoL, the score is directly proportional to overall QoL, meaning a higher SF-36 score demonstrates higher HRQoL [36].

#### 2.4.3. Pain Intensity Numerical Rating Scale (PINRS)

An 11-point Pain intensity numerical rating scale (PINRS) ranging from 0 (no pain) to 10 (worst possible pain) was used, asking participants to rate their current pain intensity [58]. The PINRS is a widely used, valid, and reliable instrument for quantifying subjective pain perception. It is simple to administer and easily understood by patients. Higher scores reflect greater pain intensity [58,59]. In this study, participants were asked to rate their current and worst levels of pain using the PINRS.

### 2.5. Translation and Cross-Cultural Adaptation Process

Following permission obtained from the copyright holder (Vaughan Keeley) [26], translation and cross-cultural adaptation of the LYMQoL-arm questionnaire into Croatian followed international guidelines [60].

#### 2.5.1. Forward Translation

Forward translation from the original English version was undertaken independently by two Croatian lymphedema specialists (A.P. and I.K.K.). They were instructed to capture the conceptual meaning of the items while employing colloquial language to ensure comprehensibility for the average patient.

#### 2.5.2. Reconciliation

Discrepancies between the two versions were critically reviewed, and a consensus was reached to produce a unified preliminary Croatian version.

#### 2.5.3. Back Translation

This version was then subjected to backward translation into English by two bilingual professional translators (T.K. and I.D.), who were blinded to the original questionnaire.

#### 2.5.4. Back Translation Review and Harmonisation

An expert committee of two professional translators and two local rehabilitation professionals fluent in English and experienced in the clinical and methodological aspects of lymphedema (physiatrists, A.P., and physiotherapist, I.K.K.), reviewed the semantic, idiomatic, and conceptual equivalence of the items and response options. Consensus was achieved on a pre-final version that aimed to preserve the meaning of the source text while expressing it in accessible lay language.

#### 2.5.5. Cognitive Debriefing

The pre-final Croatian version was pilot tested in a group of ten women with arm lymphedema of varying ages and socioeconomic backgrounds, all treated at the University Hospital Split. After completing the questionnaire, participants underwent cognitive debriefing to assess the appropriateness, cultural relevance, and clarity of the questionnaire in detail.

#### 2.5.6. Review of Cognitive Debriefing Results and Finalisation

Based on the feedback obtained, the expert committee finalised the text, resulting in the definitive Croatian version, LYMQoL-UL-CRO (available upon request from the authors upon request).

#### 2.5.7. Proofreading and Editing

The final version was proofread by all forward translators. Authors have developed a visually clear layout suitable for printing, performed the final editing and formatting

### 2.6. Reliability

#### 2.6.1. Internal Consistency

The psychometric validation of the LYMQoL-UL-CRO followed a rigorous methodological framework. Reliability was assessed through internal consistency with Cronbach’s α for all domains (Function, Appearance, Symptoms, Mood).

#### 2.6.2. Test–Retest Reliability

Participants were asked to complete LYMQoL-UL-CRO Arm twice with an interval of ten days. All participants completed the first questionnaire on the same day that limb volume measurements were taken. The retest was completed after ten days by 68 participants. The test–retest reliability was calculated using intraclass correlation coefficients derived from retest via a two-way random-effects model. Additionally, the reliability of the LYMQoL-UL-CRO was assessed by calculating the Standard Error of Measurement (SEM) and the Smallest Real Difference (SRD) for each domain.

### 2.7. Validation

#### 2.7.1. Content Validity

Content validity was explored by administering a short follow-up questionnaire to the first 47 participants after completion of the LYMQoL-UL-CRO. Six questions assessed clarity, comprehensibility, and ease of use, as well as whether any items or response categories appeared redundant.

#### 2.7.2. Criterion and Construct Validity

Construct validity was examined by assessing Spearman’s rank correlations, assessing associations between LYMQoL-UL-CRO domains and SF-36 physical and mental component summaries (PCS and MCS) as gold standards, and Association with current and worst pain intensity as measured by Numerical rating scale (PINRS) was also assessed.

Criterion validity was examined by comparing LYMQoL-UL-CRO domain scores with established measures, including the SF-36 PCS and MCS and the Numerical Rating Scale (PINRS) for current and worst pain intensity, using Spearman’s rank correlation coefficients to assess the strength and direction of associations.

#### 2.7.3. Discriminant Validity

Discriminant validity was tested by comparing LYMQoL-UL-CRO scores between participants with clinical (RVC ≥ 5%) and those with subclinical (RVC < 5%) lymphedema groups using Welch’s *t*-test.

### 2.8. Responsiveness

Participants were also asked whether the instrument adequately addressed the challenges they experienced due to BCRL, whether completion time was acceptable, and how long it took them to complete the questionnaire.

### 2.9. Factor Structure

The factor structure was examined using exploratory factor analysis (EFA) with Varimax rotation, retaining factors with eigenvalues greater than 1. The Kaiser-Meyer-Olkin (KMO) measure and Bartlett’s test of sphericity confirmed data suitability.

### 2.10. Floor/Ceiling Effects

Floor/Ceiling Effects were also evaluated by calculating the proportions of participants scoring the minimum or maximum possible values in each domain, with thresholds set at <15%.

### 2.11. Quality of Evidence for the Measurement Properties of LYMQoL

The quality of evidence for the measurement properties of the LYMQoL questionnaire in this study was evaluated using the Consensus-based Standards for the selection of health Measurement Instruments (COSMIN) methodology. Each measurement property assessed in our dataset was rated according to the COSMIN criteria for good measurement properties. Methodological quality was judged using the COSMIN Risk of Bias checklist, and ratings (sufficient, insufficient, or indeterminate) were assigned for each property based on the observed results [30,61].

### 2.12. Statistical Analysis

Statistical analysis was performed using IBM SPSS Statistics, Version 26 (IBM Corp., Armonh, NY, USA) software. Internal consistency was measured with Cronbach’s alpha to assess the reliability of each domain. Test–retest reliability was determined by having participants complete the LYMQoL-UL-CRO on two occasions, calculating the intraclass correlation coefficient (ICC) using a two-way random effects model. Construct validity was assessed by calculating correlations. For data that were not normally distributed, we used Spearman’s rank correlation coefficient, while for normally distributed data, we used Pearson’s correlation coefficient. Factor structure was examined through exploratory or confirmatory factor analysis with Varimax rotation, and the suitability of the data was checked using the Kaiser-Meyer-Olkin (KMO) measure and Bartlett’s test of sphericity. Floor and ceiling effects were assessed to ensure appropriate response distribution, with thresholds set at 15%. For group comparisons, non-parametric tests such as the Kruskal–Wallis analysis of variance, Mann–Whitney U-tests, and Welch’s *t*-test were used. Missing data were handled by multiple imputations with five imputed datasets, and the significance level was set at *p* < 0.05.

## 3. Results

### 3.1. Translation Process

The translation process is described in detail in Section 2. Only minor differences were identified across four forward translations due to the favourably simple language used in the original version. A review of the backward translation showed near complete equivalence to the original English version. The formatted final version of LYMQoL-UL-CRO is available from the authors upon request.

### 3.2. Participants’ Demographics and Treatment-Related Characteristics

Data from 87 breast cancer survivors were analysed, with an average interval of six years between completion of breast cancer treatment and study enrolment. Approximately 60% of participants had undergone mastectomy and total axillary lymph node dissection. In 54% of cases, surgery was performed on the dominant side of the body. Detailed data on participants’ sociodemographic and treatment-related characteristics are summarised in Table 1.

Data from 87 breast cancer survivors were analysed, with detailed results presented in Table 2. Participants were stratified into two subgroups based on relative volume change (RVC < 5% vs. RVC ≥ 5%). As shown in Table 2, individuals with RVC ≥ 5% were significantly older than those with RVC < 5% (60.5 ± 10.9 vs. 55.3 ± 9.8 years; *p* = 0.021) and more frequently reported swelling (85.1% vs. 53.8%; *p* = 0.001). No significant between-group differences were observed for body mass index, time since surgery, dominant arm involvement, mastectomy, axillary lymph node dissection, or adjuvant therapies (radiotherapy, chemotherapy, or endocrine therapy).

### 3.3. Reliability

#### 3.3.1. Internal Consistency

All LYMQoL-UL-CRO domains showed adequate internal consistency (α > 0.70). The Symptoms and Function domains demonstrated the highest reliability (α = 0.91 and 0.85, respectively), followed by Appearance (α = 0.85). Although the Mood domain initially showed lower internal consistency (α = 0.75), its reliability markedly improved at retest (α = 0.92), indicating temporal stability of this subscale, with detailed results presented in Table 3.

#### 3.3.2. Test–Retest Reliability

All domains show moderate reliability (ICC 0.68–0.73). The analysis shows adequate test–retest reliability, though slightly lower in the Appearance domain (Table 4).

The reliability of the LYMQoL-UL-CRO was assessed by calculating the Standard Error of Measurement (SEM) and the Smallest Real Difference (SRD) for each domain. SEM quantifies the expected variability in repeated measurements, ranging from 0.31 in the Symptoms domain to 0.45 in the Appearance domain. The SRD, indicating the minimal change required to confidently assert a true difference beyond measurement error, ranged from 0.86 in Symptoms to 1.25 in Appearance.

### 3.4. Validity

#### 3.4.1. Criterion and Construct Validity

Table 5 shows that the LYMQoL-UL-CRO Function (ρ = −0.65, *p* < 0.001) and Symptoms (ρ = −0.70, *p* < 0.001) domains have strong negative correlations with the SF-36 Physical Component Summary (PCS), while the Mood domain (ρ = −0.60, *p* < 0.001) has a moderate negative correlation with the SF-36 Mental Component Summary (MCS). The Appearance domain shows weak, non-significant correlations with SF-36 components (*p* > 0.05). These results support the construct validity of the LYMQoL-Arm.

The Symptoms domain had the strongest and statistically significant positive correlations with all PINRS variables (*p* < 0.01), indicating that higher symptom burden is associated with worse self-reported outcomes. In contrast, the Mood factor did not show significant correlations with any PINRS variable (*p* > 0.05), while the Function and Appearance factors demonstrated weaker or only partially significant associations (Table 5).

#### 3.4.2. Discriminant Validity

Discriminant validity is demonstrated in Table 6, as it compares LYMQoL-UL-CRO and SF-36 scores between clinical and subclinical lymphedema groups to show whether the questionnaire can distinguish between patients with different levels of disease.

Patients with higher RVC (≥5%) scores reported significantly worse outcomes in LYMQoL-UL-CRO Function (F = 4.23, *p* = 0.016), Symptoms (F = 5.67, *p* = 0.004), and Mood (F = 3.45, *p* = 0.035) compared to those with RVC < 5%. No significant differences were observed for Appearance or SF-36 summary components (PSC/MSC).

#### 3.4.3. Content Validity

The 47 participants all agreed that the questionnaire was simple to complete and that the response format was clear. None of the respondents thought the questionnaire was too long or included unnecessary questions. All respondents confirmed that the questionnaire addressed the challenges they encounter because of their lymphedema.

##### Responsiveness

None of the participants thought the time needed to finish the questionnaire was too long. With a minimum of two minutes and a maximum of ten, the average completion time was approximately five minutes.

### 3.5. Factor Analysis

EFA identified four factors that together explained 60.49% of the total variance: Function (34.66%), Appearance (10.41%), Symptoms (9.13%), and Mood (6.29%) (Table 7). All factor loadings exceeded the 0.3 threshold. The suitability of the data for factor analysis was confirmed by a high Kaiser-Meyer-Olkin (KMO) value of 0.89 and a statistically significant Bartlett’s test of sphericity (χ^2^ = 2104.32, *p* < 0.001).

### 3.6. Floor and Ceiling Effects

Floor effects represent the percentage of participants scoring the minimum value (1), while ceiling effects indicate those scoring the maximum value (4). All values remain below the recommended 15% threshold, demonstrating appropriate questionnaire design without significant response clustering (Table 8). The slightly elevated floor effect in the Function domain (18.2% test/15.9% retest) suggests a mild tendency toward minimal impairment reporting in physical function items, consistent with findings in chronic lymphedema populations. Retest values show improved distribution, indicating stable measurement properties.

### 3.7. Quality of Evidence for the Measurement Properties of LYMQoL

The assessment of the psychometric properties of the LYMQoL-UL-CRO questionnaire demonstrated very good characteristics in most domains. All results of the psychometric evaluation of the LYMQoL-UL-CRO, including methodological quality according to COSMIN criteria and overall quality of evidence ratings, are systematically summarised and presented in Table 9.

## 4. Discussion

The primary aim of this study was to translate and validate the Croatian version of the LYMQoL-UL questionnaire. Our findings demonstrate that the LYMQoL-UL-CRO possesses strong psychometric properties, is culturally appropriate, and captures the multidimensional impact of BCRL on HRQoL in Croatian breast cancer survivors. Furthermore, in addition to the psychometric analyses, the quality of the LYMQoL-UL-CRO measurement properties was evaluated according to COSMIN guidelines, providing a structured and internationally recognised framework for assessing PROM quality [61]. Using the COSMIN Risk of Bias checklist, the methodological quality of each measurement property was judged as adequate, and all properties were subsequently rated against COSMIN criteria for good measurement performance. The LYMQoL-UL-CRO demonstrated sufficient quality of evidence for content validity, structural validity, internal consistency, reliability, measurement error, and construct validity. The certainty of evidence was graded as very good. This comprehensive COSMIN-based evaluation further supports the robustness of the Croatian version of the LYMQoL-Arm and confirms that it meets recommended standards for use in both clinical practice and research

In the scope of our knowledge, this is the first study to assess the validity and reliability of a lymphedema-specific quality of life instrument within the Croatian healthcare context.

### 4.1. Content Validity and Cultural Adaptation

The evaluation of content validity in this study confirmed that the Croatian version preserved the conceptual integrity of the original instrument. All participants found that the LYMQoL-UL-CRO was clear, comprehensive, and easy to complete, consistent with previous findings demonstrating LYMQoL items reflect concepts relevant to women with BCRL [26,62]. No missing items or conceptual inconsistencies were identified. These findings suggest that the translation accurately reflects the linguistic and cultural context of Croatian patients. This feedback process ensured conceptual item selection across multiple domains [62]. The overall consistency of findings with previous international adaptations confirms that LYMQoL-UL-CRO remains both a conceptual and practical equivalent to the original tool [26,29,41].

### 4.2. Reliability

The physiometric properties of the Croatian version of the LYMQoL-UL-CRO closely mirror findings from previous international studies, confirming its reliability and clinical relevance [29,33,38,41,63]. High internal consistency (α > 0.75–0.91) and moderate test–retest reliability (ICC 0.68–0.73), individual consistent measurement across time, with slightly lower stability observed in the Appearance domain. Slightly reduced ICC values in this domain may be explained by day-to-day fluctuations in limb volume, particularly in milder cases [13,21,29,55]. Additionally, appearance perceptions can vary with transient swelling, mood fluctuations, or changes in body image, which naturally introduce greater variability. The chosen 10-day retest interval reflects a methodological trade-off: it is sufficiently long to minimise recall bias, yet long enough for natural symptom fluctuation to occur. Previous studies used shorter retest intervals (2–7 days), which likely reduced symptom variability and may therefore explain their comparatively higher ICC values [29,33].

Sensitivity testing further supported the instrument’s precision and suitability for detecting true changes in individual symptoms and appearance over time. Low SEM and SRD values across all domains indicate that this tool provides consistent and dependent scores upon repeated measures [64]. These results demonstrate satisfactory measurement precision and stable psychometric properties, supporting its use in clinical monitoring and follow-up.

The slightly elevated floor effect in the Function domain suggests a mild tendency toward minimal impairment reporting in physical function items, consistent with findings in chronic lymphedema populations [15,21]. However, the reduction in the floor effect at retest suggests improved distribution and supports the stability of measurement properties over time.

### 4.3. Validity

Construct and validity analysis revealed strong and theoretically consistent correlations between LYMQoL-UL-CRO and the SF-36 summary components. As expected, the Function and Symptoms domains correlated strongly and negatively with the SF-36 Physical Component Summary (PCS), while the Mood domain showed a moderate negative correlation with the Mental Component Summaries (MCS). These negative correlations are conceptually expected. Higher symptom burden or functional impairment on LYMQoL-UL-CRO necessarily reflects poorer perceived health status, resulting in lower SF-36 scores [28,65]. These relationships support the conceptual overlap between two measures and confirm that LYMQoL-UL-Cro appropriately captures both physical and psychological dimensions of HRQoL in BCRL and align with results from other cultural validations, where stronger associations with physical domains have been reported [29,33,36,37,41]. Moreover, similar results were obtained when comparing LYMQoL with other generic HRQoL instruments, such as the EQ-5D, EORTC QLQ-C30, and Nottingham Health Profile, which reinforce its construct validity and applicability across diverse populations [33,38,63].

Discriminant validity was also established as LYMQoL-UL-CRO successfully distinguished between participants with subclinical and early-clinically lymphedema (RVC < 5% vs. RVC ≥ 5%). Notably, this threshold is lower than the traditional criterion of ≥10% volume increase typically used to define clinically evident lymphedema [45,46,51]. Yet LYMQoL-UL-CRO was still able to detect meaningful differences in HRQoL. These findings highlight the instrument’s ability to capture HRQoL impairments even in relatively small but clinically important volume increases, underscoring its sensitivity in early disease stages [47,53,66].

Patients with greater RVC reported significantly worse Function, Symptoms, and Mood scores, confirming that the presence of clinically defined lymphedema meaningfully affects HRQoL. These results are consistent with previous studies showing that patients with more severe conditions experience increased arm heaviness, tightness, pain, and body image disturbance [18,28,36,67,68]. In contrast, neither perceived physical appearance nor overall health status, as assessed by the generic SF-36 questionnaire, differed significantly between groups. This finding suggests that disease-specific tools such as LYMQoL-UL-CRO are more sensitive to the subtle yet clinically meaningful impact of lymphedema on daily functioning and emotional adjustment, compared to generic instruments [28,33,63,65].

Given that LYMQoL-UL-CRO will primarily be used by rehabilitation team, its clinical utility extends beyond outcome evaluation to practical guidance for goal setting and treatment planning. The ability of this instrument to identify patients at risk of functional or emotional burden provides valuable support for clinicians’ decision-making and helps determine when intensified monitoring or intervention may be warranted [69]. By enabling a detailed understanding of symptom burden and functional limitations, this instrument contributes to the development of an individualised, patient-centred model of lymphedema management [27,28,40]. Worsening of Function or Symptom scores can guide clinicians towards initiation or intensification of Complete Decongestive Therapy, adjusting compression therapy, exercise programmes, or referring patients for psychological support when emotional or appearance concerns arise [2].

Finally, its incorporation in routine follow-up care may also inform future updates of survivorship guidelines, particularly for breast cancer survivors, a population for whom current recommendations beyond five years post-treatment remain limited [2,6,70]. Its structured use within prospective surveillance models, administered preoperatively, early postoperatively, and at regular follow-up intervals, enabling timely identification of meaningful changes that may precede detectable limb swelling [9]. While discriminant validity demonstrates an instrument’s ability to differentiate between levels of clinically defined lymphedema, further interpretation is needed to understand how these clinical indicators relate to patients’ subjective experiences.

### 4.4. Clinical Interpretation and Construct Relationships

Our results also contribute to the ongoing discussion regarding the relationship between clinical indicators and self-reported outcomes. Consistent with previous studies, HRQoL did not always correlate with objective measures such as limb volume, indicating that volume alone does not determine well-being [26,41]. Although increases in limb volume (RVC ≥ 5%) may signal lymphatic impairment [47,71,72], subjective experiences of symptoms and appearance often better reflect the true impact of lymphedema on QoL [22,26,41,55,71,73,74]. Recent studies further confirm this discrepancy, showing that limb volume only partially explains QoL variance, while subjective symptom burden is more strongly associated with functional limitations and emotional distress [75]. Our findings support this interpretation, as overall HRQoL did not differ significantly between women with higher and lower RVC values, suggesting that psychological and functional factors may play a greater role than physical swelling alone [22,26,41,55,76,77]. These results underscore the importance of individualised, patient-centred management strategies that integrate both subjective and objective assessments, as previously recommended [22,76,78]. Such an approach provides a more comprehensive understanding of patients’ needs and may enhance the precision of therapeutic decision-making.

Interpretation of our findings should also consider current shifts in breast cancer surgery. Modern axillary management has reduced BCRL incidence compared to historical cohorts [8,13].

In our study, however, around 60% of women underwent ALND, and most of them received radiotherapy, indicating that the present results are more applicable to survivors at higher baseline risk.

Despite these shifts, LYMQoL-UL-CRO remains highly relevant in modern treatment pathways where subtle early volume changes, particularly following SLND and radiotherapy, still require careful monitoring. By enabling early detection of patient-reported impairments, the instrument supports a proactive and longitudinal approach to survivorship care [2].

### 4.5. Strengths and Limitations

The major strength of this study is its novelty; it represents the first validation of disease-specific PROM for upper limb lymphedema in Croatia. The study fills both a clinical and scientific gap by providing a culturally relevant tool with proven psychometric quality. The study achieved excellent feasibility and acceptability, demonstrated by a 100% completion rate, no missing responses, and a very short average completion time consistent with previous studies. Another strength lies in the comprehensive evaluation of both reliability and validity, as well as the instruments’ proven ability to distinguish patients with different levels of disease severity, confirming their clinical utility and cross-cultural comparability. In contrast to the majority of prior research, our study incorporated an objective assessment of limb volume alongside subjective outcomes. Nevertheless, several limitations should be acknowledged. First, the cross-sectional design limits the ability to detect changes over time and prevents any causal interpretation between lymphedema severity and quality-of-life outcomes. Additionally, the retrospective and single-centr nature of the study may have introduced selection bias and further restricted causal inference. The effective sample size was relatively small due to the 4:1 particle-to-participant ratio; a larger ratio would have improved the precision of the analyses. Moreover, because our institution is both a tertiary referral center and is the only regional facility providing specialised lymphedema care, the potential for spectrum bias exists, although comprehensive referral patterns may partially mitigate this. The LYMQoL instrument is also region-specific, designed primarily for upper-limb lymphedema, and therefore does not capture quality-of-life issues related to lymphedema at other anatomical sites. Future research should include larger, more diverse patient populations and employ longitudinal designs to assess sensitivity to change and responsiveness to interventions over time.

## 5. Conclusions

The Croatian LYMQoL-Arm questionnaire demonstrated strong psychometric properties, including high reliability, robust construct, and discriminant validity, and a clear factor structure. These findings confirm that the Croatian version is a reliable, sensitive, and culturally appropriate tool for assessing HRQoL in women with breast cancer-related lymphedema. Its implementation in clinical and research practice can enhance individualised patient assessment, enable early detection of disease impact, and contribute to improved survivorship care and long-term outcomes.

Importantly, our findings reinforce that volume measurements alone are insufficient to capture the full extent of patient burden. Objective changes should always be interpreted in conjunction with LYMQoL-UL-CRO scores to obtain a more accurate understanding of patients’ lived experiences. Furthermore, symptom clusters such as pain, heaviness, tightness, and functional restriction may provide more clinically meaningful guidance for tailoring physiotherapy interventions than absolute differences in limb volume. By integrating both objective and patient-reported measures, clinicians can deliver more precise, responsive, and patient-centred lymphedema management.

## Figures and Tables

**Table 1:** Characteristics of participants (n = 87).

Variable	Measure/Category	Value
Age (years)	M ± SD ^1^	58.47 ± 8.95
BMI ^2^ (kg/m^2^)	M ± SD	27.74 ± 5.64
Marital status n (%)	Married	54.84%
	Single	18.28%
	Widowed	15.05%
Education level n (%)	High school diploma	50.54%
	Bachelor’s degree	21.51%
	Primary school	13.98%
Household income n (%)	<1000 €	38 (41.8%)
	1000–2000 €	31 (34.1%)
	>2000 €	16 (17.6%)
Years since the BC ^3^ surgery	M ± SD	6.25 ± 5.33
Type of the BC surgery; n (%)	Breast conserving	41.4%
	Mastectomy	58.6%
Lymph node removal; n (%)	SLND ^4^	35 (40.2%)
	ALND ^5^	52 (59.8%)
Radiotherapy; n (%)	Yes	75.9%
	No	24.1%
Chemotherapy; n (%)	Yes	60.9%
	No	39.0%

Note:^1^ mean ± standard deviation; ^2^ Body Mass Index; ^3^ Breast Cancer; ^4^ Sentinel Lymph Node Biopsy; ^5^ Axillary Lymph Node Biopsy.

**Table 2:** Basic characteristics of participants by lymphedema subgroup: clinical lymphedema (RVC ≥ 5%) and subclinical lymphedema (RVC < 5%).

Variable	RVC ^1^ < 5% (*n =* 40)	RVC ≥ 5% (*n* = 47)	*p*-Value
Age (years); M ± SD	55.3 ± 9.8	60.5 ± 10.9	0.021 *
BMI ^2^ (kg/m^2^); M ± SD	26.8 ± 4.9	28.7 ± 5.2	0.084
Time since surgery (years); M ± SD	6.2 ± 4.8	7.9 ± 5.4	0.137
Dominant arm affected; n (%)	13 (33.3%)	19 (40.4%)	0.505
Mastectomy; n (%)	16 (41.0%)	25 (53.2%)	0.247
ALND ^3^; n (%)	19 (48.7%)	31 (66.0%)	0.096
Therapy; n (%)			
Radiotherapy; n (%)	30 (76.9%)	38 (80.9%)	0.648
Chemotherapy; n (%)	25 (64.1%)	34 (72.3%)	0.417
Endocrine therapy; n (%)	27 (69.2%)	35 (74.5%)	0.573
Self-reported swelling; n (%)	21 (53.8%)	40 (85.1%)	0.001 **

Note: ^1^ Relative Volume Change; ^2^ Body Mass Index; ^3^ Axillary Lymph Node Dissection, * *p* < 0.05; ** *p* < 0.01.

**Table 3:** Cronbach’s alpha coefficients for the LYMQoL Arm.

LYMQoL ^1^ Domain	Cronbach’s α	Cronbach’s α Retest
Function	0.850	0.791
Appearance	0.846	0.855
Symptoms	0.907	0.901
Mood	0.748	0.921

Note:^1^ Lymphedema Quality of Life Questionnaire.

**Table 4:** The reliability of the LYMQoL-UL-CRO was assessed through test–retest consistency.

LYQMoL Domain	Test (M ± SD) ^1^	Retest (M ± SD)	ICC ^2^	*p*-Value	SEM ^3^	SRD ^4^
Function	2.1 ± 0.7	2.0 ± 0.6	0.71	<0.01	0.38	1.05
Appearance	2.0 ± 0.8	1.9 ± 0.7	0.68	<0.01	0.45	1.25
Symptoms	2.2 ± 0.6	2.1 ± 0.5	0.73	<0.01	0.31	0.86
Mood	2.0 ± 0.7	1.9 ± 0.6	0.69	<0.01	0.39	1.08
Global	6.3 ± 2.1	6.1 ± 1.9	0.70	<0.01	1.15	3.19

Note: ^1^ mean ± standard deviation; ^2^ ICC—Intraclass Correlation Coefficient (two-way random effects model for absolute agreement); ^3^ Standard Error of Measurement = SD × √(1 − ICC); ^4^ Smallest Real Difference = 1.96 × √2 × SEM.

**Table 5:** Correlation between LYMQoL-UL-CRO domains and SF-36 and PINRS measurements.

LYQMoL ^1^ Domain	SF-36 ^2^ PSC ^3^	SF-36 MSC ^4^	PINRS ^5^ Current	PINRS Worst
Function	−0.65 *	−0.20	0.29 *	0.29 *
Appearance	−0.30	−0.15	0.22 *	0.25 *
Symptoms	−0.70 *	−0.25	0.37 **	0.41 **
Mood	−0.40	−0.60 *	0.15	0.17

Note: ^1^ Lymphedema Quality of Life Questionnaire; ^2^ Short Form (36) Health Survey; ^3^ Physical Component Summary; ^4^ Mental Component Summary; ^5^ Numerical Rating Scale; * *p* < 0.05; ** *p* < 0.01.

**Table 6:** Comparison of LYMQoL-UL-CRO Factors and SF-36 Scores by lymphedema subgroup: clinical lymphedema (RVC ≥ 5%) and subclinical lymphedema (RVC < 5%).

Domain	RVC ^1^ < 5% (*n* = 40)	RVC ≥ 5% (*n* = 47)	F-Statistic	*p*-Value
LYQMoL ^2^-Arm Function	1.23 ± 0.45	1.89 ± 0.52	4.23	0.016 *
LYQMoL-Arm Appearance	1.57 ± 0.31	1.72 ± 0.41	1.12	0.331
LYQMoL-Arm Symptoms	1.95 ± 0.61	2.78 ± 0.67	5.67	0.004 *
LYQMoL-Arm Mood	1.82 ± 0.39	2.45 ± 0.58	3.45	0.035 *
SF-36 ^3^ PSC ^4^	42.1 ± 8.2	38.5 ± 7.6	2.98	0.087
SF-36 MSC ^5^	48.3 ± 9.1	44.7 ± 8.9	1.75	0.189

Note: ^1^ Relative Lymphedema Change; ^2^ Lymphedema Quality of Life Questionnaire; ^3^ Short Form (36) Health Survey; ^4^ Physical Component Summary; ^5^ Mental Component Summary; * *p* < 0.05.

**Table 7:** Factor Analysis of the LYMQoL-UL-CRO.

LYMQoL ^1^ Arm	Function	Appearance	Symptoms	Mood	Communality
LQ ^2^ 1a	0.36	−0.19	0.39	0.03	0.45
LQ1b	0.34	0.28	0.54	0.12	0.63
LQ1c	0.62	0.15	0.43	0.02	0.68
LQ1d	0.44	0.03	0.62	0.09	0.67
LQ1e	0.76	−0.02	0.22	0.09	0.60
LQ1f	0.85	0.12	0.04	0.14	0.77
LQ1g	0.38	0.07	0.59	0.07	0.65
LQ1h	0.87	−0.01	0.06	0.11	0.76
LQ2	0.20	0.39	0.47	0.21	0.60
LQ3	0.23	0.40	0.49	0.27	0.63
LQ4	0.12	0.86	0.23	−0.07	0.82
LQ5	−0.01	0.85	0.15	0.11	0.81
LQ6	−0.04	0.85	0.06	0.06	0.73
LQ7	0.06	0.73	0.25	0.20	0.71
LQ8	0.04	0.47	0.13	0.17	0.42
LQ9	0.06	0.21	0.73	0.14	0.64
LQ10	0.05	0.21	0.82	0.12	0.78
LQ11	0.02	0.22	0.82	0.13	0.78
LQ12	−0.04	0.22	0.79	0.33	0.81
LQ13	0.07	0.50	0.55	0.33	0.76
LQ14	0.12	0.38	0.41	0.55	0.68
LQ15	0.11	0.16	0.21	0.77	0.73
LQ16	0.14	0.24	0.16	0.73	0.71
LQ17	0.15	0.10	0.15	0.86	0.79
LQ18	0.01	0.26	0.14	0.72	0.70
LQ19	−0.07	−0.09	0.16	0.88	0.82
LQ20	−0.04	−0.03	−0.02	0.30	0.29

Note: ^1^ Lymphedema Quality of Life Questionnaire; ^2^ Lymphedema Question.

**Table 8:** Floor and ceiling effects across LYMQoL-UL-CRO domains in both questionnaires.

LYQMoL ^1^ domain	Floor (%)	Ceiling (%)	Floor (%)	Ceiling (%)
	Test		Retest	
Function	18.2	9.1	15.9	6.8
Appearance	12.7	5.4	9.8	4.1
Symptoms	8.3	3.9	6.2	2.7
Mood	10.6	4.8	7.5	3.6

Note: ^1^ Lymphedema Quality of Life Questionnaire.

**Table 9:** Psychometric properties of LYMQoL-UL-CRO according to Consensus-based Standards for the selection of health Measurement INstruments (COSMIN) checklist.

Measurement Property	COSMIN ^1^ Criteria/Requirement	LYMQoL-UL-CRO ^2^ Results	Quality Assessment
Content Validity	Item relevance, clarity, coverage of all aspects of the construct	All items are relevant and clear; the construct is thoroughly covered	Very good
Structural Validity	Factor analysis performed; KMO ^3^ > 0.7; significant Bartlett test	KMO ^3^ = 0.876; 4-factor model confirmed by EFA ^4^	Very good
Internal Consistency	Cronbach’s alpha ≥ 0.70 for all domains	Function α = 0.85, Appearance α = 0.85, Symptoms α = 0.91, Mood α = 0.75	Very good
Reliability (Test–Retest)	ICC ^5^ > 0.70 for all domains	Function ICC = 0.71, Appearance ICC = 0.68, Symptoms ICC = 0.73, Mood ICC = 0.69 (CI ^6^ 0.61–0.78, *p* < 0.01).	Good
Measurement Error	SEM ^7^ and SRD ^8^ reported	SEM (Function: 0.38, Appearance: 0.45, Symptoms: 0.31, Mood: 0.39, Global: 1.15); SRD (Function: 1.05, Appearance: 1.25, Symptoms: 0.86, Mood: 1.08, Global: 3.19)	Adequate
Criterion Validity	Strong correlations with gold standards (SF-36 ^9^ PCS ^10^, MCS ^11^, PINRS ^12^)	Function r = −0.65 (*p* < 0.001), Symptoms r = −0.70 (*p* < 0.001) with SF-36 PCS; Mood r = −0.60 (*p* < 0.001) with SF-36 MCS; correlations with PINRS ranged from 0.22 to 0.41 (*p* < 0.05) for all domains except Mood	Very good
Construct Validity	Correlations with related constructs	Confirmed by Spearman’s rank correlations with SF-36 PCS, MCS, and PINRS	Very good
Discriminant Validity	Differentiation between clinical and subclinical lymphedema	Significant differences in Function (F = 4.23, *p* = 0.016), Symptoms (F = 5.67, *p* = 0.004), Mood (F = 3.45, *p* = 0.035) domains	Very good
Responsiveness	Usability, completion time, floor and ceiling effects	Mean completion time approx. 5 min; floor effects 6.2–18.2%; ceiling effects 2.7–9.1% across domains	Very good
Cross-Cultural Validity	Equivalence of forward/back translation and adaptation	Near-complete equivalence, with minor linguistic adjustments	Very good

COSMIN—^1^ COnsensus-based Standards for the selection of health Measurement INstruments; ^2^ LYMQoL—Lymphedema Quality of Life Questionnaire; UL—Upper Limb; CRO—Croatian version; ^3^ KMO—Kaiser-Meyer-Olkin measure of sampling adequacy; ^4^ EFA—Exploratory Factor Analysis; ^5^ ICC—Intraclass Correlation Coefficient; ^6^ CI—Confidence Interval; ^7^ SEM—Standard Error of Measurement; ^8^ SRD—Smallest Real Difference; ^9^ SF-36—Short Form Health Survey—36 Items; ^10^ PCS—Physical Component Summary; ^11^ MCS—Mental Component Summary; ^12^ PINRS—Numeric Rating Scale; F—F-statistic; *p*—probability value.

## Data Availability

The data presented in this study are available on request from the corresponding author.

## Abbreviations

The following abbreviations are used in this manuscript:

TLA	Three-letter acronyms
LYMQoL	Lymphedema Quality of Life Questionnaire
SF-36	Short-Form Health Survey
PI-PINRS-11	Pain Intensity Numerical Rating Scale
RVC	Relative Volume Change
BCRL	Breast Cancer-Related Lymphedema
BMI	Body Mass Index
QoL	Quality of Life
HRQoL	Health-Related Quality of Life
UL	Upper Limb
CRO	Croatian
PROM	Patient Reported Outcome Measure
SF-36	Short Form Health Survey—36 Items
PCS	Physical Component Summary
MCS	Mental Component Summary
ICC	Intraclass Correlation Coefficient
CI	Confidence Interval;
KMO	Kaiser-Meyer-Olkin measure of sampling adequacy;
LQ	LYMQoL Question
SD	Standard Error of Measurement
α	Cronbach Alpha
EORTC QLQ C30	European Organisation for Research and Treatment of Cancer Quality of Life Questionnaire Core 30.
EQ 5D	EuroQol 5-Dimensions questionnaire
PINRS	Numerical rating Scale
F	F-statistics
*p*	Probability value
SRD	Smallest Real Difference
